# The Role of Nursing Diagnoses in Enhancing Prognostic Accuracy in Home‐Based Cancer Care: Insights From a Retrospective Cohort Study

**DOI:** 10.1111/jocn.17821

**Published:** 2025-05-19

**Authors:** Luca Bertocchi, Andreina Saba, Angelo Dante, Monica Guberti, Sandra Consolini, Stefania Tortella, Dorothy Jones, Cristina Petrucci, Loreto Lancia

**Affiliations:** ^1^ Department of Health, Life, and Environmental Sciences University of L'Aquila L'Aquila Italy; ^2^ The Marjory Gordon Program for Clinical Reasoning and Knowledge Development, William F. Connell School of Nursing Boston College Chestnut Hill Massachusetts USA; ^3^ Local Health Unit Reggio Emilia Reggio Emilia Italy

## Abstract

**Aims:**

To (1) describe the characteristics of patients with advanced cancer receiving home‐based care, (2) identify the nursing diagnoses associated with 6‐month mortality and (3) explore the predictive power of nursing diagnoses on 6‐month mortality for patients with an advanced cancer diagnosis.

**Background:**

Nursing diagnoses have been shown to capture the complexity of patients' experiences and the specific nursing care related to patients' responses to illness, including increased mortality risk. However, there is a lack of studies investigating the relationship between nursing diagnoses and mortality among cancer patients receiving home‐based care.

**Design:**

Retrospective cohort study.

**Methods:**

Between July 2021 and June 2023, patients with advanced cancer were consecutively admitted to a home‐based care service. Medical data, prognostic indexes and nursing assessment data, including nursing diagnoses from NANDA International, assigned during the first home visit, were extracted from patient health records. Survival analysis was performed over the first 6 months using the Kaplan–Meier method and Cox proportional hazards model.

**Results:**

Among 344 enrolled patients, the most frequent nursing diagnoses were chronic pain and constipation. The 45.9% of patients died at home within 6 months after discharge. Multivariate Cox regression identified a Palliative Prognostic Index ≥ 5, palliative status, terminal phase of illness and two nursing diagnoses—imbalanced nutrition: less than body requirements and death anxiety—as significant predictors of 6‐month mortality.

**Conclusions:**

Survival in advanced cancer patients receiving home care was primarily predicted by the terminal phase of illness, Palliative Prognostic Index, palliative status and two specific nursing diagnoses: imbalanced nutrition: less than body requirements and death anxiety.

**Reporting Method:**

The Strengthening the Reporting of Observational Studies in Epidemiology (STROBE) guidelines were adopted in this study.

**Patient or Public Contribution:**

No Patient or Public Contribution.

**Summary**

- What does this paper contribute to the global clinical community?
  ○ Incorporating nursing assessment framework and diagnoses into health records alongside prognostic indexes enhances the accuracy of the focus of nursing and links diagnosis to survival predictions and patient complexity assessments in home‐based cancer care.
  ○ Nursing patient complexity, combined with clinical prognostic factors, aids mortality prediction, promoting patient‐centered palliative care and interdisciplinary collaboration.
  ○ Use of standardised nursing terminologies in electronic records helps identify high‐risk patients early, articulate nursing contributions to collaborative care models and optimise palliative interventions and care resources.

## 1 Introduction

Cancers represent the second‐leading cause of death worldwide (Ritchie et al. 2018; World Health Organization 2023a). The increase in life expectancy of the population in general and the consequent higher prevalence of cancers and other chronic degenerative diseases represent a global issue (OECD 2023). Within this complex scenario, nurses provide comprehensive, patient‐centered care to patients diagnosed with cancer from the initial diagnosis to treatment, and during the late stage of the disease by relieving patients from suffering through palliative care (World Health Organization 2023b). In the last decades, a shift in resource use from hospital to home‐based care services is supporting this increased demand and ensuring the sustainability of the national health service (OECD 2023). Home‐based care is regulated by national and international guidelines and represents a new opportunity for cancer patients to address the challenges of illness, supported by a multidisciplinary team, especially in end‐of‐life and palliative care. Mortality rates for patients diagnosed with advanced cancer are reported to be highly variable; a typical period of 12 months before death is often indicative of palliative care needs, a benchmark endorsed by global quality standards (Owusuaa et al. 2022).

Palliative care aims to enhance the quality of life for individuals and families dealing with life‐threatening illnesses by identifying and addressing physical, psychosocial, and spiritual issues to prevent and alleviate suffering (World Health Organization 2023b). Early palliative care can have a beneficial impact on the patient's quality of life and help reduce the negative outcomes including patient and caregiver distress, increased hospitalisations, accesses to the emergency department, admissions to the intensive care unit and even survival (de Camargo et al. 2022; Pergolizzi et al. 2020; Colombet et al. 2019; Bakitas et al. 2015).

Patients living with advanced cancer require a bio‐psycho‐social‐spiritual approach to multidisciplinary care that is focused on ‘taking care of the person as a whole’ rather than ‘focusing only on the illness’ (bio‐medical paradigm). This holistic, person‐centered approach to care can be incorporated effectively in organisations evaluating nursing care delivery with a sufficient level of detail about the person and the meaning associated with the response to cancer. This approach includes use of a wholistic approach to patient assessment data, generation of a clinical judgements (i.e., nursing diagnoses), and nursing interventions that are critical to determining a nursing response to complex care requirements and nursing expertise needed to achieve high‐quality, safe, cost‐effective patient care outcomes.

## 2 Background

Nurses represent the largest category of professional health workers (OECD 2023) and play a key role in the multidisciplinary team in providing a holistic approach to respond to terminally ill patients in palliative home care (Chow and Dahlin 2018). Despite the importance of nursing care delivery to patients with advanced cancer, the collection and communication of nursing data is often unstructured and unorganised, making it impossible to effectively articulate a focus of care or evaluate the actual impact of nursing on patient outcomes. Use of standardised nursing terminologies, such as NANDA‐International (NANDA‐I) classification, is recommended but frequently overlooked in healthcare documentation systems (Bertocchi et al. 2023). These terminologies facilitate communication of nursing judgements and decisions regarding holistic human experiences and responses to health and illness (Rodríguez‐Suárez et al. 2023; Bertocchi et al. 2023; Fennelly et al. 2021; Zhang et al. 2021; Macieira et al. 2018; Othman et al. 2020). Nursing diagnoses within the NANDA‐I classification serve as a measure of patient nursing complexity (D'Agostino et al. 2017) and nursing dependency (Sanson et al. 2019). Nursing diagnoses capture and synthetize the nurses' clinical judgement about patients' and family's human responses regarding health conditions or life processes (Herdman et al. 2021). Nursing data can complement medical data to better describe patient's complexity (Sanson et al. 2017). The nursing diagnoses within the NANDA‐International (NANDA‐I) classification are used worldwide and represent the most developed and utilised standardised nursing terminologies recognised by the American Nurses Association (Bertocchi et al. 2023; Zhang et al. 2021; Fennelly et al. 2021; Macieira et al. 2018).

A comprehensive review of the literature demonstrates that using nursing diagnoses can help identify hospitalised patients at higher risk of death (Bertocchi et al. 2023; Sanson et al. 2019; Juve‐Udina et al. 2017). In addition, nursing diagnoses may contain infomarkers (i.e., nonbiological data cues of the human state that are extracted from electronic health records using different techniques) that identify patients near the end of life for whom it would be appropriate to shift the trajectory of care goals to palliative care (Yao et al. 2015).

An accurate prediction of factors associated with mortality is necessary for clinical, organisational and ethical reasons, especially in helping to avoid harm, discomfort and inappropriate therapies in vulnerable patients (Glare et al. 2008). A variety of scales and classification systems have been described in the literature that suggest a systematic approach to determining patient complexity and prognosis in palliative care patients (Grant et al. 2021; Glare et al. 2008), but nursing data have not been included in these systematic approaches to date. Studies that document nursing diagnoses on admission found the diagnoses to be an indicator of nursing complexity and an independent predictor of important patient outcomes, such as mortality and length of stay, mainly in institutionalised patients (Bertocchi et al. 2023; Zeffiro et al. 2020; D'Agostino et al. 2019; Sanson et al. 2019; Castellan et al. 2016). However, to the best of the authors knowledge, no studies have been previously conducted to investigate the relationship between nursing diagnoses and mortality in adults with advanced cancer in home‐based care settings. For this reason, the aims of this study were to (1) describe the characteristics of advanced cancer patients receiving home‐based care, (2) identify the nursing diagnoses associated with 6‐month mortality and (3) explore the predictive power of nursing diagnoses on 6‐month mortality.

## 3 Methods

### 3.1 Design

A retrospective cohort study was undertaken to answer the study aims. The research report was written according to Strengthening the Reporting of Observational Studies in Epidemiology (STROBE) guidelines (von Elm et al. 2007) (see Appendix S1).

### 3.2 Setting

The study was conducted in an onco‐haematology home‐based care nursing service of ‘blinded’, a North Italian District. Nurses working in the home‐based care team provide care to approximately 350 patients with cancer per year. The goal of the team is focused on ensuring personalised care, based on individualised nursing care plans. The frequency of the nursing home visits ranged from once per week to daily, depending upon the patient's overall health and response to their health/illness conditions and the amount of nursing care required to address the patient's problems.

Nurses collaborate with a multidisciplinary team, composed of physicians, psychologists and nurse assistants constituting the Palliative Care Network. They provide integrated home‐based care to terminally ill patients. This multidisciplinary team considers the patient and caregiver(s) as the centre of the care process, from the assessment to the implementation of interventions, to the evaluation of accomplishing mutual goals.

### 3.3 Inclusion and Exclusion Criteria

The clinical records of all terminal patients consecutively admitted in the onco‐haematology home‐based care service from July 1, 2021, to June 30, 2022, were reviewed for inclusion in the study. Records of all patients followed up within a maximum period of 6 months were included. Patients were excluded from the analysis if the domiciliary care was stopped for a change of residence (*n* = 8) since data about their final outcomes at 6 months were not retrievable. Considering the exploratory nature of the study and the lack of previous data in the same setting, the calculation of sample size was not undertaken.

### 3.4 Endpoints, Variables and Sources of Data

The main endpoint of the study was the patients' 6‐month mortality from the time of their first home visit. The secondary outcome was the care status at 6 months from the first home visit (still in care, died at home, died in hospice or died in the hospital).

To fulfil the aims of the study, the following variables were also obtained: (1) Socio‐demographic characteristics (sex and age); (2) medical data assigned at the first home visit: [main *ICD‐10* medical diagnosis, palliative care service activation and level of complexity of the integrated home care service assigned by the general practitioner (GP) (Ministero della Salute 2006)]; (3) prognostic indexes (see Section 3.5); (4) number and type of NANDA International nursing diagnoses assigned at the first visit.

The data sources included the local electronic health records of the local health unit (ADI‐Web), and the nursing care plans for each patient. The nursing care plan is an electronic record containing information related to nursing care provided to patients and their families.

### 3.5 Prognostic Indexes Used to Evaluate Function in Advanced Cancer Patients

Three different prognostic tools were used by nurses: the Italian versions of the Australian‐modified Karnofsky Performance Status (AKPS), the Palliative Prognostic Index (PPI), and the Phase of Illness.

- The AKPS (Veronese 2020) is a tool able to assess the functional decline of cancer patients and provide prognostic information (Ferrucci et al. 2007; Maltoni et al. 2005). The AKPS score ranges from 0 (dead) to 100 (perfect health). Higher scores indicate a better ability of the patient to carry out their daily living activities (Abernethy et al. 2005). A low performance status is considered a reliable prognostic factor to predict short‐term survival. An AKPS score of less than 70% is an early indicator for palliative care, while a score ≤ 50% suggests a terminal stage of illness (de Camargo et al. 2022).
- The PPI (Canzani et al. 2018) relies on the assessment of five variables: the oral intake, the presence or absence of edema, dyspnea at rest, delirium and performance status. The PPI generates a numerical score ranging from 0 to 15 and three prognostic categories. Scores > 6/15 points predict a survival time less than 3 weeks. Scores ranging from 5 to 6/15 predict a survival time less than 6 weeks, while scores ≤ 4/15 indicate that the expected survival time is higher than 6 weeks (Kao et al. 2014; Stone et al. 2008).
- The Phase of the illness (Veronese 2020) provides four categories highlighting the trajectory of the disease (stable, unstable, deterioration and dying).

### 3.6 Reducing and Eliminating Bias

To minimise the *selection* bias, all the health records and nursing care plans of patients admitted in the home‐based care service during the study period were reviewed. The *information* bias related to the accuracy of data collection during the nursing assessment was limited, guaranteeing the participation of nurses in a 40‐h educational program concerning nursing diagnoses and prognostic indexes. In addition, data reported by nurses during the first home visit were shared with the nursing teams to confirm their completeness and reliability. Gordon's Eleven Functional Health Patterns (Gordon 1994) was used as a standardised framework to assess functional and dysfunctional patterns. The identified nursing problems were then discussed and encoded by using the NANDA‐I taxonomy according to the NANDA‐I handbook 2021–2023 (Herdman et al. 2021). This approach encouraged interprofessional communication and provided a consistent and systematic documentation of patient history. By strengthening data collection practices and promoting standardised assessments, the validation of patient problems and nursing diagnoses was enhanced, ultimately improving diagnostic accuracy.

### 3.7 Data Analysis

Descriptive analyses were used to describe both the characteristics of the sample and care provided to patients by home care nurses. Categorical variables were described through frequencies and percentages. Continuous variables were expressed as mean (standard deviation), median (IQR) and min‐max. The normality distribution of the data was visually assessed using histograms, boxplots, Q‐Q plots and explored using the Kolmogorov–Smirnov or Shapiro–Wilk tests (Polit and Beck 2021).

The survival analysis was performed using the Kaplan–Meier estimator. Both the Log‐Rank test and Hazard Ratio (Cox regression) were calculated by dichotomising variables according to the following criteria. Level of complexity was assigned by the GP (low complexity vs. medium and high complexity); PPI (survival shorter than 6 weeks vs. survival longer than 6 weeks); AKPS (≤ median value vs. > median value); number of nursing diagnoses (median value). The Cox regression model was performed using a blockwise approach. The first block was constituted by medical data assigned at the first home visit and prognostic indexes significantly associated with mortality probability in Kaplan–Meieranalysis. The same procedure was used for the second block represented by the number and the types of nursing diagnoses. Finally, the third block included both Block 1 and Block 2. The statistical significance level was fixed at *p* ≤ 0.05. All data were analysed using STATA version 17.0 (Stata, College Station, TX).

### 3.8 Ethical Considerations

The study was conducted in accordance with the ethical principles outlined in the Declaration of Helsinki. All data extracted from patient records were de‐identified, and each anonymized record was assigned a unique, sequential numerical code before inclusion in the database. The Ethics Committee of Local Health Unit was consulted and provided an opinion stating that formal ethical approval was not deemed necessary, as the data were anonymized at the source (letter 2022‐09.12).

## 4 Results

### 4.1 Characteristics of the Study Population

Overall, 352 patients were admitted to the home‐based care service during the year of observation. As reported in Table 1, over half of the study sample consisted of females (*n* = 174, 50.6%). The mean age was 76.4 (SD 12.3) years [median 79 (69–85), min‐max 25–100]. The most frequently occurring malignant neoplasms, according to the ICD‐10 classification, were those of the digestive and respiratory systems (33.4% and 22.1%, respectively). Based on the judgement of the GP, over three‐quarters of the patients were in a medium complexity of care status (77.6%) and required palliative care (75.3%). The mean AKPS score was 51.1/100 (SD 16.2) (median 50 (40–60), min max 10–90), while the PPI score was ranging mainly ≤ 4/15 (59.3%). Finally, most of the patients (41.0%) were in a deterioration phase of the illness.

**TABLE 1:** Characteristics of patients at the first home visit (*N* = 344).

Variables	Mean (SD)	*n*	%
Age (years)	76.4 (12.3)		
Sex			
Female		174	50.6
Male		170	49.4
Malignant neoplasm type:^a^			
Digestive organs		115	33.4
Respiratory/intrathoracic organs		76	22.1
Blood		32	9.3
Breast		29	8.4
Genital organs		29	8.4
Urinary tract		18	5.2
Others		45	13.1
Level of complexity assigned by the GP			
Low complexity (I level)		34	9.9
Medium complexity (II level)		267	77.6
High complexity (III level)		43	12.5
Palliative care status assigned by the GP			
Yes		259	75.3
No		85	24.7
Australian Karnofsky Performance Status/100	51.1 (16.2)		
Palliative Prognostic Index/15			
Survival longer than 6 weeks (PPI 0–4)		204	59.3
Survival shorter than 6 weeks (PPI 5–6)		83	24.1
Survival shorter than 3 weeks (PPI ≥ 7)		57	16.6
Phase of illness			
Deteriorating		141	41.0
Unstable		111	32.3
Terminal		50	14.5
Stable		42	12.2
Number nursing diagnoses on the first visit	3.6 (1.2)		
First 20 actual NANDA‐I nursing diagnoses			
00133—Chronic pain		162	47.1
00011—Constipation		95	27.6
00032—Ineffective beathing pattern		49	14.2
00134—Nausea		41	11.9
00002—Imbalanced nutrition: less than body requirements		39	11.3
00132—Acute pain		33	9.6
00093—Fatigue		30	8.7
00026—Excess fluid volume		29	8.4
00085—Impaired physical mobility		24	7.0
00103—Impaired swallowing		22	6.4
00128—Acute confusion		21	6.1
00196—Dysfunctional gastrointestinal motility		19	5.5
00312—Adult pressure injury		19	5.5
00095—Insomnia		18	5.2
00046—Impaired skin integrity		14	4.1
00108—Bathing self‐care deficit		14	4.1
00088—Impaired walking		13	3.8
00126—Deficient knowledge		12	3.5
00147—Death anxiety		12	3.5
00045—Impaired oral mucous membrane		12	3.5

Abbreviations: GP, general practitioner; SD, Standard deviation.

^a^ ICD‐10 Classification of medical diagnoses.

### 4.2 Nursing Diagnoses in Home‐Based Cancer Care

A total of 1236 nursing diagnoses, corresponding to an average of 3.6 (SD 1.2) (median 3.5(3–4), min max 1–8) nursing diagnoses per patient, were documented. Overall, 105 nursing diagnoses were selected at least once: 64 actual diagnoses (61.0%), 27 (25.7%) risk diagnoses, 12 (11.4%) health promotion diagnoses and 2 (1.9%) syndromes. The most frequent actual nursing diagnoses attributed to patients were ‘chronic pain’ (47.1%), ‘constipation’ (27.6%) and ‘ineffective beathing pattern’ (14.2%) (Table 1). The most frequent actual nursing diagnoses attributed to patients' family were ‘compromised family coping’ (45, 13.1%), and ‘disabled family coping’ (19, 5.5%). Finally, the most prevalent risk diagnoses were ‘risk for infection’ (95; 27.6%), ‘risk for adult fall’ (27, 7.8%) and ‘risk for bleeding’ (25, 7.3%).

### 4.3 Endpoints

Overall, 264/344 (76.7%) patients died during the 6‐month follow‐up period. The majority of patients died at home (158; 45.9%). The others died in hospice (*n* = 59; 17.2%) and hospitals (*n* = 47; 13.7%) while about one‐fifth of the sample (*n* = 80; 23.3%) were still in care (Table 2). The average survival time for all patients was 79.2 days (SD 68.5) (median 52; min‐max 1–180).

**TABLE 2:** Endpoints at 6 months (180 days).

Endpoints	Mean (SD)	*n*	%
Six‐month mortality		264	76.7
Status of patients at the end of the six‐month follow‐up			
Died at home		158	45.9
Died in the hospice		59	17.2
Died in the hospital		47	13.7
Still in care		80	23.3
Survival time (days)	79.2 (68.5)		

Abbreviation: SD, standard deviation.

### 4.4 Six‐Month Survival Analysis

**FIGURE 1:**
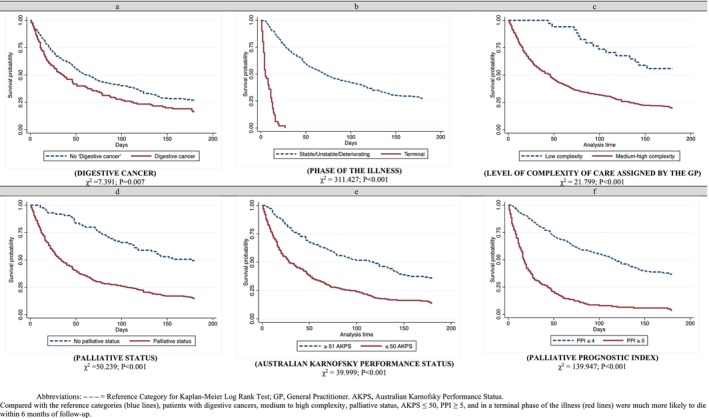
Six‐month survival analysis based on medical data and prognostic indexes assigned at the first home visit. [Colour figure can be viewed at wileyonlinelibrary.com]

A statistically lower survival likelihood was found in patients diagnosed with digestive cancers, those in the terminal phase of the illness, patients with medium/high complexity status or palliative status, and those who had an AKPS score ≤ 50 and a PPI score ≥ 5 (Figure 1).

**FIGURE 2:**
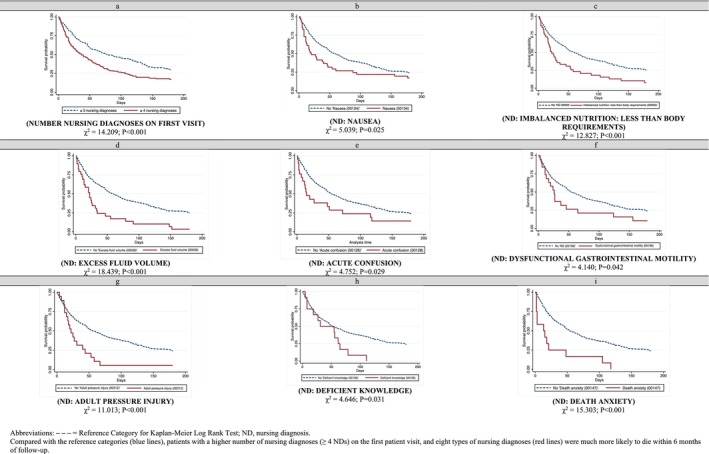
Six‐month survival analysis based on actual nursing diagnoses assigned at the first home visit. [Colour figure can be viewed at wileyonlinelibrary.com]

This outcome was also observed in patients who received four or more nursing diagnoses during the first visit, as well as those who received the following nursing diagnoses: ‘nausea,’ ‘imbalanced nutriton: less than body requirements,’ ‘excess fluid volume,’ ‘acute confusion,’ ‘dysfunctional gastrointestinal motility,’ ‘adult pressure injury,’ ‘deficient knowledge’ and ‘death anxiety’ (Figure 2). Data related to variables not significantly associated with survival analyses are reported in Supporting Information 2.

As highlighted in the blockwise Cox regression analysis (Table 3), testing the first group of variables revealed that having a PPI ≥ 5, being in the terminal phase of the illness, and having a palliative status significantly increased the likelihood of mortality at 6 months.

**TABLE 3:** Factors predicting six‐month mortality: Multivariate Cox regression analysis.

	HR	95% CI		*p*
		Lower	Upper	
Medical data and prognostic indexes assigned at the first home visit (Group 1)				
Palliative Prognostic Index (≤ 4^a^ vs. ≥ 5)	2.462	1.803	3.363	< 0.001
Phase of illness (deteriorating/stable/unstable^a^ vs. terminal)	8.049	5.270	12.292	< 0.001
Palliative status assigned by the GP (no^a^ vs. yes)	2.074	1.389	3.097	< 0.001
Digestive cancer (no^a^ vs. yes)	1.172	0.909	1.512	0.221
Level of complexity assigned by the GP (low^a^ vs. medium/high)	1.185	0.629	2.233	0.599
Australian Karnofsky Performance Status (≥ 50^a^ vs. ≤ 50)	1.247	0.931	1.669	0.138
Nursing diagnoses assigned at the first home visit (Group 2)				
Number nursing diagnoses at the first visit (≤ 3^a^ vs. ≥ 4)	1.299	1.002	1.683	0.048
00002—Imbalanced nutrition: less than body requirements (no^a^ vs. yes)	1.938	1.347	2.787	< 0.001
00026—Excess fluid volume (no^a^ vs. yes)	2.393	1.587	3.610	< 0.001
00128—Acute confusion (no^a^ vs. yes)	1.767	1.089	2.867	0.021
00147—Death anxiety (no^a^ vs. yes)	3.566	1.952	6.515	< 0.001
00134—Nausea (no^a^ vs. yes)	1.392	0.949	2.041	0.090
00196—Dysfunctional gastrointestinal motility (no^a^ vs. yes)	1.631	0.976	2.724	0.062
00312—Adult pressure injury (no^a^ vs. yes)	2.061	1.247	3.406	0.005
00126—Deficient knowledge (no^a^ vs. yes)	1.826	0.994	3.354	0.052
Medical data, prognostic indexes and nursing diagnoses assigned at the first home visit (Group 3)				
Palliative Prognostic Index (≤ 4^a^ vs. ≥ 5)	2.384	1.734	3.278	< 0.001
Phase of illness (deteriorating/stable/unstable^a^ vs. terminal)	9.318	5.887	14.749	< 0.001
Palliative status assigned by the GP (no^a^ vs. yes)	2.102	1.394	3.168	< 0.001
Digestive malignant neoplasm (no^a^ vs. yes)	1.092	0.840	1.419	0.512
Level of complexity assigned by the GP (low^a^ vs. medium/high)	1.066	0.557	2.038	0.847
Australian Karnofsky Performance Status (≥ 50^a^ vs. ≤ 50)	1.212	0.897	1.638	0.210
Number nursing diagnoses at the first visit (≤ 3^a^ vs. ≥ 4)	1.100	0.841	1.439	0.486
00002—Imbalanced nutrition: less than body requirements (no^a^ vs. yes)	1.997	1.380	2.890	< 0.001
00026—Excess fluid volume (no^a^ vs. yes)	1.439	0.939	2.205	0.095
00128—Acute confusion (no^a^ vs. yes)	0.617	0.363	1.049	0.074
00147—Death anxiety (no^a^ vs. yes)	2.463	1.332	4.552	0.004
00134—Nausea (no^a^ vs. yes)	1.243	0.814	1.898	0.314
00196—Dysfunctional gastrointestinal motility (no^a^ vs. yes)	1.502	0.875	2.580	0.140
00312—Adult pressure injury (no^a^ vs. yes)	1.383	0.824	2.322	0.220
00126—Deficient knowledge (no^a^ vs. yes)	1.074	0.585	1.974	0.818

*Note:* Group 1: PPI ≥ 5, terminal phase of the illness, and a palliative status significantly increased 6‐month mortality probability. Group 2: A higher number of nursing diagnoses identified during the first visit, the presence of five different types of nursing diagnoses significantly increased 6‐month mortality probability. Group 3: Adding Block 1 to the Block 2, there was a significant improvement in prediction of mortality (χ^2^ = 29.588; *p* < 0.001). In addition to medical data assigned at the first home visit and prognostic indexes significant in Block 1 (PPI, terminal phase of the illness, palliative status), two nursing diagnoses (imbalanced nutrition: less than body requirements, death anxiety) were confirmed to be predictors of mortality.

Abbreviation: GP, General Practitioner.

^a^ Reference category for multivariate cox‐regression analysis.

**FIGURE 3:**
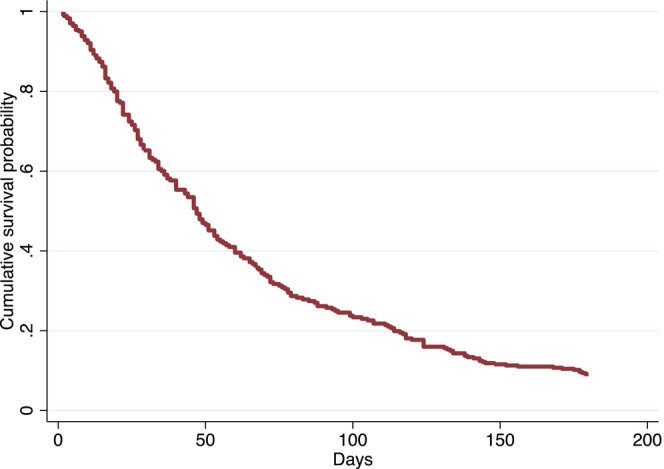
Cumulative survival probability of the Cox proportional hazards regression model. [Colour figure can be viewed at wileyonlinelibrary.com]

Testing the second group of variables showed that having four or more nursing diagnoses during the first visit, as well as receiving the nursing diagnoses ‘imbalanced nutrition: less than body requirements,’ ‘excess fluid volume,’ ‘acute confusion’ and ‘death anxiety,’ were significantly associated with an increased risk of mortality. Finally, when the nursing diagnoses from the second group were added to the variables in the first group, having a PPI ≥ 5, being in the terminal phase of the illness, having a palliative status, and receiving the nursing diagnoses ‘imbalanced nutrition: less than body requirements’ and ‘death anxiety’ continued to confirm their predictive power for 6‐month mortality. The cumulative survival probability is reported in Figure 3.

## 5 Discussion

In line with the WHO cancer report (World Health Organization 2023a), patients included in this study primarily suffered from digestive and respiratory cancers (33.4% and 22.1%, respectively). Most patients were elderly and in a deteriorating clinical condition, requiring intensive and continuous nursing care as well as frequent medical attention (de Camargo et al. 2022). Specifically, the average number of nursing diagnoses per patients (3.6) indicates a moderate nursing complexity according to the literature (Bertocchi et al. 2023). The most frequently recorded nursing diagnosis was ‘chronic pain,’ with a prevalence of 47.7%. This result is consistent with the literature, where pain is one of the most frequent symptoms in patients suffering from terminal cancer, with a prevalence ranging from 11% to 84% depending on setting, method of symptom ascertainment, selection of patients and treatment modality (Franks et al. 2000). Effective pain management poses a significant challenge in caring for terminally ill cancer patients. Through accurate pain assessment and the implementation of both pharmacological and non‐pharmacological interventions, the nursing process can be instrumental in reducing pain intensity and improving patient comfort (Lowey 2020; Morikawa et al. 2023).

Another relevant nursing diagnosis was ‘constipation’. This condition in terminal cancer patients, with a prevalence ranging from 30% to 90%, can be attributed to the use of opioids for pain management, reduced mobility, insufficient fluid and food intake and the effects of cancer therapies themselves (Dzierżanowski and Mercadante 2022). Considering that constipation can be causes of distress for patients with cancer and that its compliances can increase nursing burden and risk of hospitalisation, it is essential that home nurses are adequately prepared to manage this condition through evidence‐based interventions (Larkin et al. 2018). In this study, the use of the nursing process provided a holistic approach to assess both physical and psychological human responses to health and illness. The reported nursing diagnoses offered new data to guide care and enhance evidence‐driven outcomes even during the care of vulnerable populations. This approach is essential to ensure the quality and comfort of care. In fact, nursing diagnoses define the health problems within the exclusive competence of nursing that can influence both patient and organisational outcomes (Sanson et al. 2017). Through accurate pain and constipation assessment and management, the nursing process enables tailored interventions that address the complex needs of terminal cancer patients, enhancing quality of life and overall care experience.

As regard the endpoint of the study, most of the included patients died at home during the 6 months after the first home visit. These data reflect the quality of the home‐based care service, which meets patients' preference to receive palliative care at home during their final stages of life.

The core findings of this study identified PPI ≥ 5, terminal phase of illness, palliative status, imbalanced nutrition: less than body requirement, and death anxiety as predictors of 6‐month mortality.

The PPI is a tool used to assess the functional status of patients in palliative care, considering factors such as oral intake, presence or absence of edema, dyspnea at rest, delirium and performance status (Canzani et al. 2018). A PPI score of 5 or higher indicates significantly reduced physiological functionality, suggesting that the patient has a poorer prognosis and a higher risk of mortality (Tavares et al. 2018).

The terminal phase of illness is another predictive factor highlighted in the analysis. This phase is characterised by a rapid decline in the patient's condition, with symptoms becoming more severe and challenging to manage. Patients in this phase are generally near the end of life, with death likely within days (Mather et al. 2018). Being in this phase substantially increases the likelihood of 6‐month mortality, as confirmed by the analysis.

Palliative status reflects the overall nature of the care received by patients, focusing on relief of physical and psychological suffering and improving quality of life rather than curing the disease (Wantonoro et al. 2022). In advanced palliative status, the primary focus is on the patient's comfort and emotional support. Emotional symptoms, pain, fatigue and emotional distress are identified as the main symptoms of palliative care patients (Dobrina et al. 2014). This status indicates a limited prognosis and a high probability of short‐term mortality.

Two nursing diagnoses were shown to be predictors of six‐month mortality: ‘imbalanced nutrition: less than body requirements’ and ‘death anxiety.’ Focusing on the added value of nursing assessment data may enable nurses to address these problems earlier and promote patient comfort, for example by alleviating death anxiety.

Malnutrition is a major problem in cancer patients and a well‐known risk factor for adverse clinical outcomes in several settings (Sanson et al. 2018; Bilgin and Gozum 2018). Poor nutritional status is associated with worse survival outcomes in cancer patients (Hamaker et al. 2021; Glare et al. 2008). Therefore, inadequate nutrition should be carefully monitored by nurses, and loss of appetite should be thoroughly explored (Brunner et al. 2022; Sanson et al. 2018; Bilgin and Gozum 2018). Early identification and assessment of malnutrition should bring attention to the multidisciplinary team, and nutritional support should be proposed (Cotogni et al. 2021). An optimal nutritional status is important to improve the quality of life in advanced cancer patients. However, if the person is terminal and comfort measures are all that the patient desires, a less vigorous approach to nutritional assessment may be indicated (Worcester et al. 1991). In this case, it is essential to acknowledge the patient's autonomy and respect their choices and decisions regarding end‐of‐life care, ensuring a dignified and meaningful death aligned with their wishes.

Regarding ‘death anxiety’, which this study identified as a predictor of 6‐month mortality, there is accumulating evidence suggesting an association between this clinical condition and worse survival in cancer patients (Henson et al. 2020; Chen et al. 2015; Mitchell et al. 2011). Specifically, an increased level of anxiety has been reported during the year before death in cancer patients (Magill et al. 2022). In this context, nurses are in a key position to further assess the patients, understand them as individuals and grasp the meaning of the entire experience within the patient's life and what is important to them. Anxiety could be a manifestation of specific fears, such as fear of death and loss of family (Newman et al. 2008). The role of nurses in assessment and monitoring is essential to communicate information to the multidisciplinary team and implement strategies to improve the quality of life and reduce these unpleasant psychological symptoms. Unlike the medical model, which typically centers on disease and symptoms, nursing care emphasises the patient's responses to illness. This approach may uncover more patient‐centered expressions of living with chronic illness—such as death anxiety—rather than focusing solely on clinical symptoms like pain. It also facilitates the management of both physical and emotional responses to enhance overall quality of life.

This study identifies the valuable contributions of nursing—specifically the use of a nursing assessment framework and nursing diagnoses—in recognising responses and experiences manifested by patients during chronic illness and their potential influence on determining a critical outcome such as 6‐month mortality. This could be explained by nursing's unique holistic approach to the patient experience, including the meaning and response to illness. Nurses are in a position to understand the patient as a whole and see illness as part of a manifestation of that whole. They promote healing and can describe a problem as a response to illness, including preparation for death. The nursing assessment of the patient and the generation of a nursing diagnosis emphasise a holistic approach to the patient and their experience. This information can be shared with the multidisciplinary palliative care team, with the common goal of improving the quality of life for these frail and vulnerable patients. The integration of nursing knowledge and the use of standardised nursing data, along with medical data, should be seen as essential elements in enhancing the team's overall understanding of patient complexity. Additionally, nursing data used as infomarkers, together with other prognostic indexes, could help identify palliative care subjects from data repositories for big‐data (Yao et al. 2015), allowing them to decide where they prefer to receive care. The assessment of nursing diagnoses can help anticipate the shift needed to a palliative care trajectory (Yao et al. 2015), leading to positive outcomes related to the early identification of palliative care patients (Bakitas et al. 2015). Further studies could validate and promote evidence‐based practice, including new NANDA‐I taxonomy in the end‐of‐life period. For example, by including end‐of‐life in the time axis of NANDA‐I, as already suggested by some authors (Bragança et al. 2021), or by introducing an end‐of‐life care syndrome with the inclusion of the most prevalent nursing diagnoses in end‐of‐life stages.

### 5.1 Strength and Limitations

This study has both limitations and strengths. Specifically, data were collected using convenience sampling from a single geographical region (Italy). Although the results are largely consistent with previous studies, they should be interpreted with caution when generalising to other countries. Moreover, while this study represents the first attempt to document the impact of nursing diagnoses on 6‐month mortality in cancer patients receiving home‐based palliative care, no directly comparable studies are available. This limitation affects the ability to draw broader conclusions from the findings. Furthermore, the study's overarching aim whether—to prolong life through curative treatment or to enhance quality of life while living and dying with advanced cancer was—not explicitly addressed. Nursing care focused on promoting healing, such as pain management, nutritional support and alleviating death anxiety, may have contributed to a peaceful death by ensuring patients remained comfortable in their final days. Future research should aim to clearly define the goals of both nursing and medical care and integrate standardised nursing assessment frameworks to enhance data consistency and improve outcome analysis.

The strengths of this work include its support for identifying potential predictors of 6‐month mortality in advanced cancer patients receiving home‐based care. Additionally, it advocates for the use of nursing diagnoses in practice, encourages the adoption of standardised languages for documenting the patient experience and establishes a clear link between nursing contributions and patient outcomes.

## 6 Conclusion

Cancer patients receiving home‐based care in this study primarily had digestive and respiratory cancers. Most were elderly and in a deteriorating clinical condition. On average, each patient had 3.6 nursing diagnoses, with ‘chronic pain’ and ‘constipation’ being the most frequently recorded. The majority of patients included in the study died at home within 6 months of the initial home visit. The key findings identified several predictors of 6‐month mortality: a PPI score ≥ 5, terminal phase of illness, palliative status, imbalanced nutrition (less than body requirement) and death anxiety.

This study demonstrated that nursing diagnoses, in conjunction with other clinical data, are valuable for predicting mortality among cancer patients receiving home‐based care. Additionally, the nursing data reported provide useful insights into the focus of home‐based care for this population. However, further research is needed to explore other patient outcomes, such as quality of life, as well as organisational outcomes, including nursing care costs and workload, in advanced cancer patients receiving home‐based care.

## 7 Relevance to Clinical Practice

The findings from this study suggest significant implications for nursing practice, especially for advanced cancer patients receiving home‐based care. Nursing diagnoses can influence predictive considerations regarding patient outcomes, with some diagnoses demonstrating predictive power for mortality when combined with reliable prognostic indexes. By identifying predictive factors associated with patients' responses to illness, this study may assist healthcare providers in better understanding and anticipating patient issues, thereby facilitating the development of more effective care plans tailored to the specific needs of advanced cancer patients in home‐based settings. The study highlights the importance of nursing diagnoses, raising awareness of their unique contribution to managing terminally ill patients and guiding clinical decision‐making. By emphasising nursing diagnoses, this research underscores the role of nursing in end‐of‐life care, focusing on quality of life, preparation for dying and ensuring a meaningful death for patients and their families. This approach underscores the essential role of nursing in enhancing well‐being and comfort in non‐curative scenarios.

Overall, this study underscores the importance of recognising and documenting nursing diagnoses. Such practices can significantly advance a comprehensive, patient‐centered approach to care, potentially leading to improved outcomes for patients and families facing terminal cancer. The insights from this study can inform policymakers and organisational leaders, advocating for the integration of standardised nursing data into health records and promoting a holistic understanding of cancer patients' experiences in end‐of‐life care. Systematic inclusion of nursing diagnoses in electronic health records and large databases, combined with other clinical prognostic data, could provide a robust strategy for more accurate mortality prediction and estimation of patient complexity in advanced cancer patients receiving home‐based care. The study offers strong potential for advancing a humanistic healing nursing model by exploring health, healing and the experience of living and dying with advanced illness. It highlights the value of a standardised nursing assessment framework, the accuracy of nursing diagnoses, and the role of nursing in promoting healing, influencing mortality outcomes and supporting a peaceful death.

## Author Contributions

L.B. was involved in conceptualization, investigation, data curation, writing – original draft and writing – review and editing. A.S. was involved in conceptualization, investigation, data curation, formal analysis, writing – original draft, writing – review and editing. A.D. was involved in conceptualization, data curation, methodology, formal analysis, writing – original draft, writing – review and editing, supervision, project administration and validation. M.G. was involved in conceptualization, investigation, data curation and writing – original draft. S.C. was involved in conceptualization, investigation, data curation and writing – original draft. S.T. was involved in conceptualization, investigation, data curation and writing – original draft. D.J. was involved in conceptualization, data curation and writing – review and editing. C.P. was involved in conceptualization, methodology, writing – review and editing, supervision and validation. L.L. was involved in conceptualization, methodology, formal analysis, writing – review and editing, supervision and validation. All authors read and agreed to the final version of the manuscript.

## Conflicts of Interest

The authors declare no conflicts of interest.

## Supporting information


Appendix S1.

## Data Availability

The data supporting the findings of this study are available from the corresponding author upon reasonable request.
