# Supplementary material for: The Role of Nursing Diagnoses in Enhancing Prognostic Accuracy in Home‐Based Cancer Care: Insights From a Retrospective Cohort Study
Source: J Clin Nurs. 2025 May 19;35(1):229–41. doi: 10.1111/jocn.17821 (PMC12667005; doi:10.1111/jocn.17821)
Supplement: Supplementary file 1 — Appendix S1. [file JOCN-35-229-s001.docx]

**Supporting information 2.** Six-month survival analysis data of not significant socio-demographics/clinical variables and nursing diagnoses

| **Variables** | | **Log rank (χ^2^)** | **p-value** |
| --- | --- | --- | --- |
| Age (≥ 71* vs. ≤ 70 years) | | 0.240 | 0.625 |
| Sex (male* vs. female) | | 0.260 | 0.610 |
| Malignant neoplasm (no* vs. yes) | |  |  |
|  | Respiratory/ intrathoracic organs | 0.979 | 0.322 |
|  | Blood | 1.953 | 0.162 |
|  | Breast | 1.416 | 0.234 |
|  | Genital organs | 1.816 | 0.178 |
|  | Urinary tract | 0.000 | 0.985 |
|  | Others | 0.253 | 0.615 |
| First 20 actual nursing diagnoses (no* vs. yes) | |  |  |
|  | 00133 - Chronic pain | 0.275 | 0.600 |
|  | 00011 - Constipation | 1.128 | 0.288 |
|  | 00032 - Ineffective beathing pattern | 0.580 | 0.446 |
|  | 00132 - Acute pain | 0.223 | 0.637 |
|  | 00093 - Fatigue | 0.318 | 0.537 |
|  | 00085 - Impaired physical mobility | 0.736 | 0.391 |
|  | 00103 - Impaired swallowing | 0.756 | 0.385 |
|  | 00095 - Insomnia | 0.122 | 0.727 |
|  | 00046 - Impaired skin integrity | 2.829 | 0.093 |
|  | 00108 - Bathing self-care deficit | 2.368 | 0.124 |
|  | 00088 - Impaired walking | 0.385 | 0.535 |
|  | 00045 - Impaired oral mucous membrane | 0.963 | 0.326 |

Abbreviations: * Reference Category for Kaplan-Meier Log Rank Test; GP, General Practitioner.
